# Modestly Elevated Serum Procalcitonin Levels in Patients with Rheumatoid Arthritis Free of Active Infection

**DOI:** 10.3390/medicina56100545

**Published:** 2020-10-17

**Authors:** Khai-Jing Ng, Hui-Chun Yu, Hsien-Yu Huang Tseng, Chia-Wen Hsu, Ming-Chi Lu

**Affiliations:** 1 Division of Allergy, Immunology and Rheumatology, Dalin Tzu Chi Hospital, Buddhist Tzu Chi Medical Foundation, Dalin, Chiayi 62247, Taiwan; khaijingcool88@gmail.com; 2 Department of Medical Research, Dalin Tzu Chi Hospital, Buddhist Tzu Chi Medical Foundation, Dalin, Chiayi 62247, Taiwan; junvsusagi@gmail.com (H.-C.Y.); david_hthy@hotmail.com (H.-Y.H.T.); chiawen0114@yahoo.com.tw (C.-W.H.); 3 School of Medicine, Tzu Chi University, Hualien City, Hualien 97071, Taiwan

**Keywords:** rheumatoid arthritis, procalcitonin, disease activity index, infection, disease activity

## Abstract

*Background and objectives*: To investigate the serum procalcitonin (PCT) levels among patients with rheumatoid arthritis (RA) without active infection compared with healthy controls and to understand the relationship of PCT with RA disease activity, and treatment received by patients. *Materials and Methods*: Patients aged 20 years and above with clinician-confirmed diagnosis of RA and healthy volunteers were included during regular outpatient visits, and those with active infection symptoms and signs were excluded. RA disease activity was measured using the Disease Activity Score-28 for Rheumatoid Arthritis with erythrocyte sedimentation rate (DAS28-ESR). Medications received by the patients were also recorded. *Results*: A total of 623 patients with RA and 87 healthy subjects were recruited in this study. The mean PCT were significantly higher in patients with RA (6.90 ± 11.81 × 10^−3^ ng/mL) compared with healthy controls (1.70 ± 6.12 × 10^−3^ ng/mL) (*p* < 0.001) and the difference remained statistically significant after adjusting for age and sex. In addition, multiple linear regression analysis showed that a lower rank-transformed PCT serum level was significantly correlated with the use of biologics (*p* = 0.017) and a high DAS28-ESR score (*p* = 0.028) in patients with RA. *Conclusion*: Patients with RA have a significantly higher serum PCT levels compared with healthy controls. The use of biologics and an active RA disease activity were associated with a lower level of PCT in patients with RA. Further investigation is required to determine the optimal cutoff value of PCT among patients with RA and its association with disease activity and biologic usage.

## 1. Introduction

Rheumatoid arthritis (RA) is a common inflammatory arthritis, affecting 0.5–1% of the general population worldwide. Patients with RA are often treated with conventional synthetic disease-modifying anti-rheumatic drugs (csDMARDs) or biologic DMARDs, putting them in a relatively immunocompromised status.

RA patients are at increased risk of infection, but infection is difficult to identify in RA because infection and inflammation can appear very similar. Moreover, it is critical for rheumatologists to differentiate the process of infection or inflammation in a timely fashion, especially when they are presented with toxic signs and high inflammatory markers in order to use antibiotic prescription or escalation of immunosuppressive treatment. Thus, the development of an accurate diagnostic tool is imperative to aid clinicians to promptly make critical decisions.

During the past 20 years, many studies have been conducted in critical illness settings, suggesting that serum procalcitonin (PCT) is a specific marker for detection of bacterial infection [1,2,3]. Studies have proven the use of PCT in clinical settings, particularly in initiating or discontinuing antibiotic therapy, and to monitor the response of treatment during bacterial infection [4,5,6].

Previous studies have evaluated PCT application among autoimmune and autoinflammatory diseases, and have shown good sensitivity and specificity for the diagnosis of systemic bacterial infection [7,8]. A few studies have investigated PCT in patients with RA. Sato et al. conducted a study in 118 RA patients, revealing that PCT had a high specificity (98.2%) in predicting infection process, but the sensitivity (25.8%) was low [9]. Among these patients with RA, 18 patients did not suffer from active infection [9]. Schwenger et al. showed that the PCT levels were below 0.5 ng/mL in 14 patients with active RA [10]. Whether the serum levels of PCT in patients with RA without infection could be elevated due to the inflammatory nature of the disease itself compared with controls is not yet clear. In addition, the correlation of serum PCT levels and RA disease activities with different treatments in patients with RA is still unknown. We hypothesized that patients with RA would have elevated serum PCT levels due to the inflammatory nature of the RA itself. Thus, the aim of this study was to investigate the serum PCT levels among patients with RA, but without active infection compared with the controls, and to evaluate the relationship of PCT with RA disease activity, and treatment received by patients.

## 2. Materials and Methods

### 2.1. Study Design and Study Population

All participants signed informed consent under a study protocol approved by the institutional review board of Dalin Tzu Chi Hospital, Buddhist Tzu Chi Medical Foundation (No. B10604003, 20 November 2017). The study was carried out in accordance with the Declaration of Helsinki. The blood sample was collected together in our earlier study [11]. In brief, patients aged 20 years and above, with clinician-confirmed diagnosis of RA based on the 2010 American College of Rheumatology (ACR)/European League Against Rheumatism (EULAR) criteria were included during regular outpatient visit. Control group was obtained from healthy volunteers who did not have active or major and uncontrolled chronic medical conditions evaluated by a physician. Patients with RA and controls who had active infection symptoms and signs were excluded from the study. Data were ascertained by laboratory measurements and medical records. RA disease activity was measured using DAS28-ESR. Patients receiving csDMARDs, including glucocorticoid, hydroxychloroquine, methotrexate, sulfasalazine, and leflunomide were considered as the non-biologic group, whereas those receiving etanercept, adalimumab, golimumab, abatacept, tocilizumab, tofacitinib, and rituximab were considered as the biologic group. Since the infection symptoms and signs of the patients with RA could be masked by the use of immunosuppressive drugs, medical chart review was performed and patients with active infection within one week after enrollment were excluded.

### 2.2. Measurement Serum PCT Levels

Serum samples were collected and stored at −80 °C. Procalcitonin were measured using enzyme-linked immunosorbent assay by a commercially available kit (Uscn Life Science Inc., Houston, TA, USA) according to the manufacturer’s protocol.

### 2.3. Statistical Analysis

Data were presented as mean with standard deviation or median with interquartile range, as appropriate. Student’s *t*-test or Mann–Whitney U test was used for the comparison of normally or non-normally distributed variables, respectively. Chi-square test was used for the comparison of categorical variables. As PCT was not normally distributed, we rank transformed the values of PCT and then used the ranks as the dependent variable in conventional simple and multiple linear regression analysis to determine the associations of PCT with different clinical parameters in patients with RA. All statistical analyses were performed using IBM SPSS, version 24.0 (IBM Corp, Armonk, NY, USA). A value of *p* < 0.05 was considered statistically significant.

## 3. Results

A total of 623 patients with RA and 87 healthy subjects were recruited in this study, and the demographic data are shown in Table 1.

**Figure 1:**
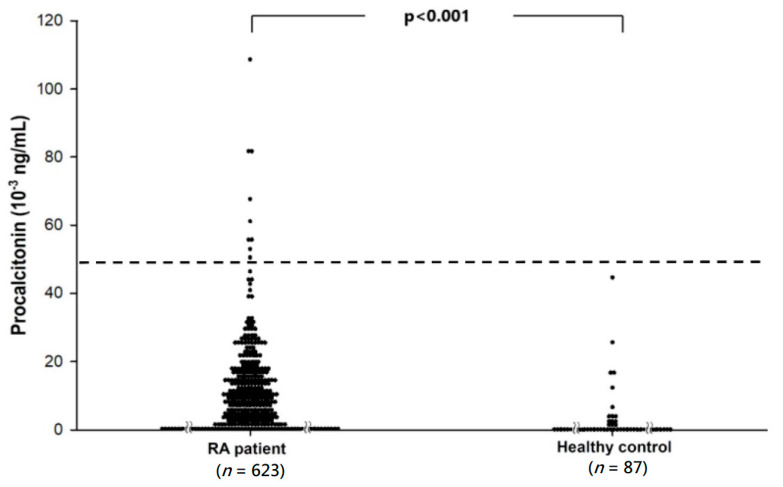
Scattered dot plot showed that mean procalcitonin was significantly higher in patients with RA (6.90 ± 11.81 × 10^−3^ ng/mL) compared with healthy controls (1.70 ± 6.12 × 10^−3^ ng/mL). The dotted line showed the cutoff value of 0.05 ng/mL, a normal cutoff value in the general population. Nine patients with RA have procalcitonin values higher than 0.05 ng/mL. Procalcitonin levels were significantly elevated in patients with RA compared with those in controls after adjusting for sex and age (*p* < 0.001).

The median PCT were significantly higher in RA patients (0.23; interquartile range (IQR) 0.00–10.56 × 10^−3^ ng/mL) compared with the controls (0.00; IQR 0.00–0.00 × 10^−3^ ng/mL), *p* < 0.001 (Figure 1). After adjusting for age and sex, the PCT levels remain significantly elevated in patients with RA (*p* < 0.001).

We performed additional subgroup analysis of serum PCT levels in patients with RA by sex, biologics usage, and RA disease activity measured by DAS28-ESR (Table 2). We found that RA patients under biologics usage (0; IQR 0.00–9.54 × 10^−3^ ng/mL) had low serum PCT levels compared to those did not used biologics (2.21; IQR 0.00–14.06 × 10^−3^ ng/mL; *p* = 0.025).

We further investigated the correlations of rank-transformed PCT levels with different clinical parameters in patients with RA. The use of biologics (*p* = 0.012) and DAS28-ESR (*p* = 0.016), but not age, sex, disease duration, CRP, csDMARD, and comorbidities, were found to be significantly and inversely correlated with serum PCT levels in patients with RA (Table 3). Results from multiple linear regression analysis also showed that a lower rank-transformed PCT serum levels remained significantly correlated with the use of biologics (*p* = 0.017) and DAS28-ESR (*p* = 0.028).

## 4. Discussion

Our study showed that patients with RA had significantly elevated serum PCT levels compared with the controls. PCT—a 116-amino acid peptide and the pre-hormone of calcitonin, firstly discovered in 1975—is normally secreted by the C cells of the thyroid in response to hypercalcemia, and has no known hormonal activity [4,9,12]. It has a half-life of 22 to 35 h (4). Patients without infection and inflammation usually have low serum PCT concentrations (<0.05 ng/mL), as all the PCT formed in C cells is converted into calcitonin [13]. In response to severe inflammation, particularly bacterial infection, PCT is released into the circulation [5]. A plasma level of ≥0.5 ng/mL is interpreted as abnormal, and indicates a diagnosis of sepsis [2,5,6]. Although it is secreted in thyroid C-cells under normal circumstances, a previous study showed that increased PCT release during severe inflammation even in thyroidectomy patients, suggesting that the production of PCT during inflammation must be outside of the thyroid glands [4].

Our study recruited a relatively large number of participants, including 201 non-biologics RA patients and 422 biologics RA patients, and revealed a significantly higher level of PCT among patients with RA when compared with healthy controls. We were surprised to find that RA patients using biologics or higher disease activity had lower serum levels of PCT, and further studies are needed to explain this association. Among them, nine RA patients were found to have a PCT level higher than 0.05 ng/mL. None were in an active infection state based on a physician’s evaluation and examination of subsequent medical records. Two were under hemodialysis, two patients had chronic obstructive pulmonary disease, two patients had chronic hepatitis, and the rest were free of comorbidities or infection. It is worth mentioning that, before using PCT level to distinguish infection and inflammation process, further studies are warranted to find out the optimal cutoff value in predicting infection process among patients with RA.

Elevated PCT levels provided a good diagnostic tool for detection systemic infection in patients with systemic autoimmune diseases [14]. Our study found that slightly but significantly elevated PCT levels in patients with RA compared to the controls. Different cutoff point of PCT had been suggested in different conditions of various infection [15]. Thus, patients with RA might need a different cutoff point of PCT in predicting an active infection process.

There are a few limitations in our study. First, we did not have the PCT levels of RA patients with an active infection process. However, during medical chart review, two of our patients with RA were excluded due to active infection within one week of enrollment, and their serum PCT levels were markedly elevated (>0.5 ng/mL). Secondly, controls and especially patients with RA might have mild infection that did not detect during enrollment by the physician. Finally, the results of CRP, ESR, and comorbidities of the heathy controls were not available.

## 5. Conclusions

Patients with RA have a significantly higher PCT compared with healthy controls. The more severe disease activity and use of biologics in patients with RA was associated with a decrease in the serum level of PCT. Further investigation is required to determine the optimal cutoff value of PCT among patients with RA and the association of disease activity and biologics usage.

## Figures and Tables

**Table 1:** Demographic characteristics of patients with rheumatoid arthritis and healthy subjects.

Variable	Rheumatoid Arthritis (*n* = 623)		Healthy Subjects (*n =* 87)		*p* Value
	*n*	%	*n*	%	
Sex					>0.999
Female	487	(78.2)	68	(78.2)	
Male	136	(21.8)	19	(21.8)	
Age, mean ± SD	62.0 ± 13.5		62.2 ± 11.7		0.909
Comorbidity	420	(67.4)			
DAS28	3.91 ± 1.34				
TJC28	5.55 ± 6.26				
SJC28	4.55 ± 4.16				
ESR (mm/hr)	19.40 ± 15.86				
PGA	34.10 ± 25.84				
CRP (mg/dL)	0.65 ± 1.24				
PCT (×10^−3^ ng/mL)					
mean ± SD	6.90 ± 11.81		1.70 ± 6.12		
median (IQR)	0.23 (0.00, 10.56)		0.00 (0.00, 0.00)		<0.001
Disease duration					
<5 years	121	(19.4)			
≥5 years	502	(80.6)			
Disease Activity					
Remission (DAS28 ≤ 2.6)	101	(16.2)			
Low (DAS28 2.6−3.2)	95	(15.2)			
Moderate (DAS28 3.2−5.1)	312	(50.1)			
Severe (DAS28 ≥ 5.1)	115	(18.5)			
Biologics	422	(67.7)			
csDMARD	581	(93.3)			
Prednisolone	477	(76.6)			
MTX	422	(67.7)			
Hydroxychloroquine	131	(21.0)			
Leflunomide	77	(12.4)			
Sulfasalazine	341	(54.7)			

*n*: number; %: percentage; SD: standard deviation; IQR: interquartile range; TJC: tender joint count; SJC: swollen joint count; PGA: patient global assessment; CRP: C-reactive protein; PCT: procalcitonin; csDMARD: conventional synthetic disease-modifying anti-rheumatic. Biologic included etanercept, adalimumab, golimumab, abatacept, tocilizumab, tofacitinib, and rituximab.

**Table 2:** Subgroup analyses of sex, biologics usage, and rheumatoid arthritis disease activity on procalcitonin levels in patients with rheumatoid arthritis (*n* = 623).

Variable	Procalcitonin (×10^−3^ ng/mL)		*p*-Value
	Median (IQR)	Mean (SD)	
Sex			0.407
Male (*n* = 136)	0.86 (0.00, 11.75)	8.50 (15.38)	
Female (*n* = 487)	0.00 (0.00, 10.38)	6.45 (10.58)	
Medication			0.025
Non-biologics (*n* = 201)	2.21 (0.00, 14.06)	7.91 (11.15)	
Biologics (*n* = 422)	0.00 (0.00, 9.54)	6.42 (12.10)	
Rheumatoid arthritis disease activity			0.542
Remission (DAS28 < 2.6) (*n* = 101)	2.16 (0.00, 13.86)	7.61 (10.65)	
Low (DAS28 2.6−3.2) (*n* = 94)	0.07 (0.00, 11.72)	7.70 (11.88)	
Moderate (DAS28 3.2−5.1) (*n* = 312)	0.34 (0.00, 10.61)	6.78 (12.16)	
Severe (DAS28 > 5.1) (*n* = 116)	0.00 (0.00, 8.42)	5.92 (11.83)	

DAS28: Disease Activity Score-28; IQR: Interquartile range; SD: standard deviation.

**Table 3:** Simple and multiple linear regression analyses of rank-transformed procalcitonin levels among patients with rheumatoid arthritis (*n* = 623).

Variable	Simple Linear Regression Analysis			Multiple Linear Regression Analysis		
	B	(CI 95%)	*p*	B	(CI 95%)	*p*
Biologics	−16.58	(−29.46, −3.69)	0.012	−15.90	(−28.90, −2.90)	0.017
Age	0.06	(−0.39, 0.51)	0.783			
Female	−5.54	(−20.19, 9.11)	0.459			
Disease duration ≥ 5 years	5.58	(−9.72, 20.87)	0.475			
DAS28	−5.53	(−10.05, −1.01)	0.016	−5.10	(−9.64, −0.55)	0.028
CRP (mg/dL)	−1.10	(−5.97, 3.78)	0.660			
csDMARD	−2.73	(−26.87, 21.41)	0.825			
Comorbidities	4.95	(−7.96, 17.86)	0.452			

DAS28: Disease Activity Score-28; CRP: C-reactive protein; csDMARD: conventional synthetic disease-modifying antirheumatic drugs.
